# Ectopic expression of the GRAS-type transcriptional regulator *NSP2* in *Parasponia* triggers contrasting effects on symbioses

**DOI:** 10.3389/fpls.2024.1468812

**Published:** 2024-10-30

**Authors:** Sultan Alhusayni, Nick Kersten, Rik Huisman, Rene Geurts, Joël Klein

**Affiliations:** ^1^ Laboratory of Molecular Biology, Cluster of Plant Development, Plant Science Group, Wageningen University, Wageningen, Netherlands; ^2^ Biological Sciences Department, College of Science, King Faisal University, Al-Ahsa, Saudi Arabia

**Keywords:** arbuscular mycorrhiza, nodulation, *Parasponia*, *NSP2*, CYCLOPS, phytoene synthase, carotenoids

## Abstract

**Introduction:**

Plants strictly control root endosymbioses with nutrient-scavenging arbuscular endomycorrhizal fungi or nodule inducing diazotrophic bacteria. The GRAS-type transcriptional regulator NODULATION SIGNALING PATHWAY 2 (*NSP2*) is a conserved hub in this process. The *NSP2*-regulated transcriptional network is instrumental in balancing nutrient homeostasis with symbiotic interactions. *NSP2* activity is modulated post-transcriptionally by a specific microRNA. Overriding this control mechanism by ectopic expression of a miRNA-resistant *NSP2* transgene enhances the symbiotic permissiveness to arbuscular endomycorrhizal fungi. Such engineered plants may possess enhanced capacities for nutrient uptake. However, the trade-off of this strategy on plant development or other symbiotic interactions, like nodulation, is yet to be fully understood.

**Method:**

We used the nodulating *Cannabaceae* species *Parasponia andersonii* as an experimental system to study the effect of ectopic *NSP2* expression. Parasponia and legumes (Fabaceae) diverged 100 million years ago, providing a unique comparative system to dissect the nodulation trait.

**Results:**

Six independent transgenic *Parasponia* lines were generated that differed in the level of *NSP2* expression in the root from 6 to 95-fold higher when compared to the empty vector control plants. Analysis of these plants revealed a positive correlation between mycorrhization and the *NSP2* expression level, as well as with the expression of the symbiosis transcription factor *CYCLOPS* and the rate-limiting enzyme in the carotenoid biosynthetic pathway *PHYTOENE SYNTHASE1* (*PSY1*). Yet ectopic expression of *NSP2* affected plant architecture and root nodule organogenesis.

**Discussion:**

This indicates a significant trade-off when leveraging *NSP2* over-expression to enhance endomycorrhization.

## Introduction

Plants explore mutualistic relationships with soil microorganisms to enhance access to essential nutrients. The endosymbiotic interactions with arbuscular mycorrhizal fungi and nodulating nitrogen-fixing bacteria like rhizobia are the most advanced examples of such ecosystem services. As these microbes are engulfed in plant cells, they largely depend on the plant’s supply of carbon sources. These intimate endosymbiotic associations necessitate stringent regulation to prevent the microbes from exploiting the plant, especially when excess exogenous nutrients are in the soil.

The GRAS-type transcriptional regulator NODULATION SIGNALING PATHWAY2 (*NSP2*) acts as a central hub integrating the plant’s nutrient homeostasis and symbiotic engagement to orchestrate adaptive responses (Quilbé and Arrighi, 2021). *NSP2* is first identified in legumes, where it is essential for rhizobium-induced nodule formation (Oldroyd and Long, 2003; Kaló et al., 2005; Heckmann et al., 2006; Ali et al., 2014; Shtark et al., 2016; Peng et al., 2021). Subsequent genome studies revealed the gene is present in many plant species and predates the evolution of angiosperms (Lauressergues et al., 2012; Cenci and Rouard, 2017). *NSP2* of different species is, to a large extent, functionally conserved, as was shown in *trans-*complementation studies of a *Lotus japonicus nsp2* nodulation mutant using *Os*NSP2** of rice (*Oryza sativa*) (Heckmann et al., 2006). In legumes and non-legumes, *NSP2* also controls mycorrhizal infection levels (Lauressergues et al., 2012; Shtark et al., 2016; Li et al., 2022). Studies in barley (*Hordeum vulgare*) suggest that *NSP2* may do so by regulating the expression of several common symbiosis signaling genes, including the transcription factor *HvCYCLOPS* (Li et al., 2022).

*NSP2* also acts as a transcriptional regulator of the strigolactone biosynthesis pathway in a nutrient status-dependent manner (Liu et al., 2011; Li et al., 2022). Strigolactones act *in planta* and *ex planta. In planta*, strigolactones function as developmental hormones and control axillary bud and lateral root outgrowth (Ruyter-Spira et al., 2011; Brewer et al., 2015; Marzec and Melzer, 2018). Upon phosphate starvation, plants exude strigolactones into their rhizosphere, affecting the microbial community composition, including the infectiveness of arbuscular mycorrhizal fungi (Schlemper et al., 2017; Kobae et al., 2018; Carvalhais et al., 2019). Together, these findings highlight the complex regulatory role of *NSP2* as a signaling hub.

*NSP2* interacts with a suite of transcription factors to control the expression of target genes. Studies in heterologous systems showed it interacts with other GRAS-type transcriptional regulators like NSP1, DELLA, REQUIRED FOR ARBUSCULAR MYCORRHIZATION1 (RAM1), and REQUIRED FOR ARBUSCULAR DEVELOPMENT1 (RAD1), and the MYB-type transcription factor INTERACTING PROTEIN OF *NSP2* (IPN2) to control nodulation and/or mycorrhization (Hirsch et al., 2009; Gobbato et al., 2012; Park et al., 2015; Xue et al., 2015; Fonouni-Farde et al., 2016; Heck et al., 2016; Jin et al., 2016; Pimprikar et al., 2016; Xiao et al., 2020). These transcriptional modules are regulated in a complex manner. Studies in rice showed that *NSP2* expression is controlled by the MYB transcription factor PHOSPHATE STARVATION RESPONSE 2 (OsPHR2), in response to phosphate starvation (Das et al., 2022; Yuan et al., 2023). Additionally, *NSP2* translation is controlled post-transcriptionally by microRNA *miR171h* (Lauressergues et al., 2012; Hofferek et al., 2014). Transcript levels of *miR171h* correlate with available phosphate concentration, suggesting that *NSP2* is controlled transcriptionally and post-transcriptionally in a nutrient-dependent manner (Hofferek et al., 2014).

To enhance sustainable agricultural productivity, reducing reliance on inorganic fertilizers is essential. This can be achieved by better utilizing the arbuscular mycorrhizal symbiosis. The ectopic expression of *NSP2* improves mycorrhization under exogenous phosphate concentrations that generally inhibit the symbiosis, suggesting it represents a potential biotechnological strategy to enhance nutrient uptake by the plant (Li et al., 2022; Isidra-Arellano et al., 2024; Yuan et al., 2023). However, at this stage, it remains elusive what the trade-off of such a strategy will be on plant development or other symbiotic interactions, like nodulation.

Studies in the legume models showed that *NSP2* overexpression had no effect on nodulation (*Medicago truncatula*) or resulted in clustering of nodules on the root though not affecting the total number of nodules (*L. japonicus*) (Lauressergues et al., 2012; Murakami et al., 2013). We used the nodulating species *Parasponia andersonii* (*Parasponia*) as a comparative experimental system complementary to legumes to study the effect of ectopic *NSP2* expression. The nodulation trait in *Parasponia* (Cannabaceae) and legumes (Fabaceae) share an evolutionary origin but diverged soon after (~100 million years ago) (van Velzen et al., 2019). *Parasponia* nodules have a different ontogeny when compared to legumes (Behm et al., 2014) Nevertheless, the same core set of symbiotic genes is used to establish nitrogen-fixing root nodules, including *NSP2* (van Velzen et al., 2018; van Zeijl et al., 2018; Bu et al., 2020; Rutten et al., 2020; Alhusayni et al., 2024). *Parasponia* is amenable for efficient *Agrobacterium tumefaciens-*mediated transformation, giving T0 transgenic plantlets within ~3 months that can subsequently be propagated vegetatively (Wardhani et al., 2019). We generated independent transgenic *Parasponia* lines that differ in the level of *NSP2* expression from 6 up to 95-fold compared to the empty vector control plants. Analysis of these plants revealed a correlation between mycorrhization and the level of expression of *NSP2*, the transcription factor *CYCLOPS*, and all genes in the biosynthetic pathways required for the formation of strigolactones. Yet ectopic expression of *NSP2* affected plant shoot development and root nodule organogenesis.

## Results

### *Parasponia *NSP2** ox lines have a root and shoot branching phenotype

*Parasponia Pan*NSP2** was studied previously, revealing it represents a single-copy gene essential for rhizobium-induced nodulation (van Zeijl et al., 2018). To enable ectopic expression of a stable *Pan*NSP2** transcript, we first identified the putative *miR171h* target site (Lauressergues et al., 2012). Such a putative site was identified in the coding region and subsequently removed by introducing synonymous DNA mutations (**Supplementary Figure S1A**). An additional alteration was included in the *Pan*NSP2** coding region, which may enhance expression stability; the insertion of an intron (**Supplementary Figure S1B**) (Feike et al., 2019). Ultimately, two *miR171h* resistant *Pan*NSP2** versions (m*NSP2*) were used for *Parasponia* transformation, a construct with and one without an engineered intron, both driven by the constitutive *L. japonicus UBIQUITIN1* promoter (*pLjUBQ1*) (**Supplementary Figure S1B**) (Maekawa et al., 2008).

**Figure 1:**
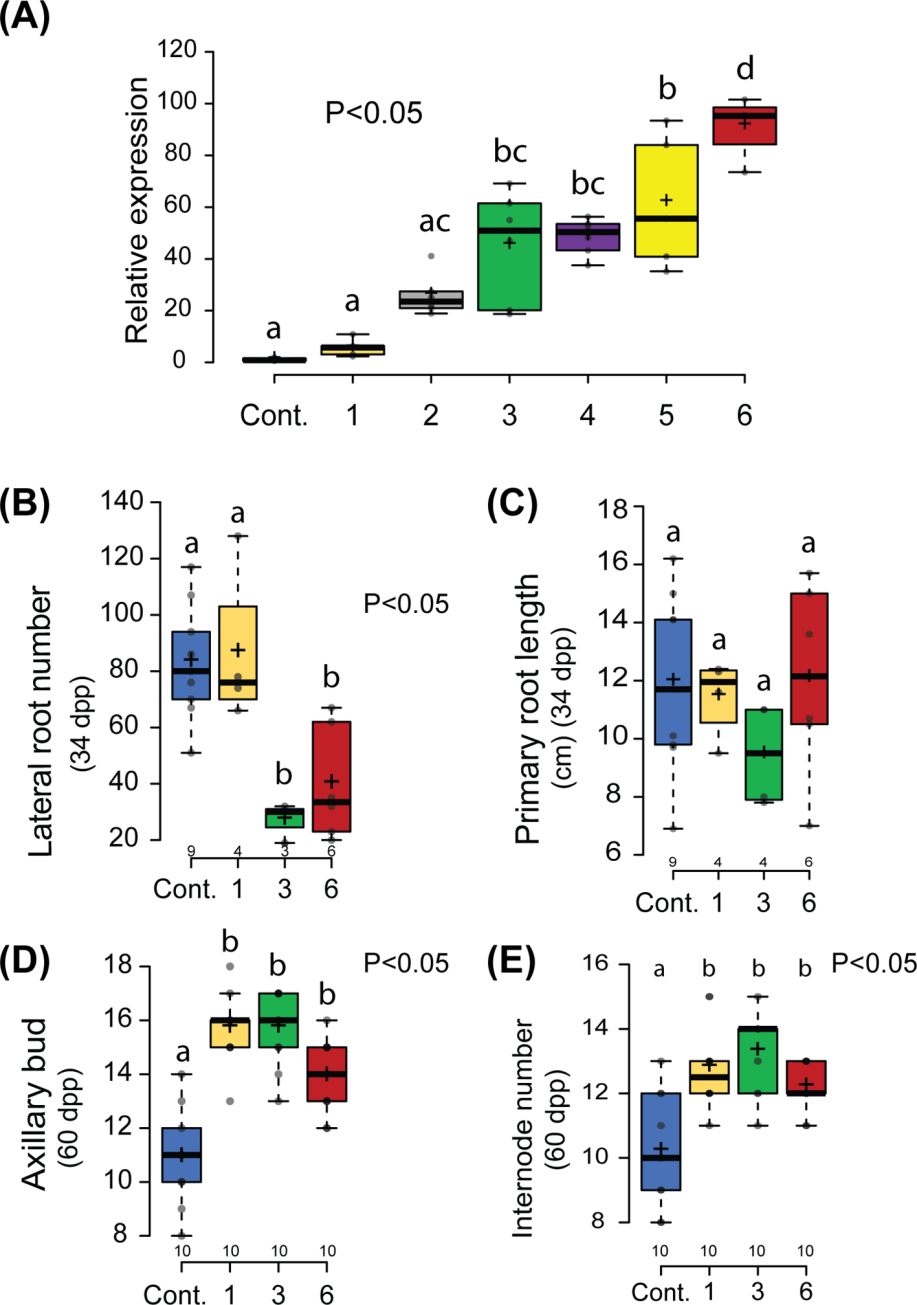
Non-symbiotic phenotype of *Parasponia* m*NSP2ox* lines. **(A)** Relative expression of *NSP2* in root tissue of 6 independent lines compared to an empty vector control line (Cont.). m*NSP2ox* lines 1 to 3 contain the transgene construct *pLjUBQ1:mPanNSP2intron*, whereas lines 4 to 6 carry *pLjUBQ1:mNSP2*. Expression was measured using qPCR (n=3). **(B, C)** Quantification of the number of lateral roots **(B)** and primary root length **(C)** of control and transgenic lines 1,3, and 6 *in vitro* grown 34 days post planting (dpp). **(D, E)** Quantification of number of lateral shoot branching **(D)** and internode **(E)** of m*NSP2ox* lines 1, 3, and 6 at 60 days post planting (dpp). Different letters indicate significant differences (p<0.05) between these lines as determined by One-way ANOVA in combination with Tukey’s *post hoc* test). All data are displayed in box plots, showing data points, the median, and the interquartile range (IQR).

Six transgenic *Parasponia* lines were selected, displaying a gradient level of *Pan*NSP2** ectopic expression ranging from a 6 to 95-fold increase in root tissue (**Figure 1A**). In the shoots of these transgenic lines, m*NSP2* was also expressed; in tissues where *Pan*NSP2** transcripts are normally not detected (**Supplementary Figure S2A**). Noteworthy, lines possessing the m*NSP2*intron construct generally have a lower transgene expression when compared to constructs possessing m*NSP2* with only an adapted *miR171h* target site.

To analyze whether morphogenic phenotypes are associated with m*NSP2* ectopic expression in *Parasponia*, we selected lines 1, 3, and 6 that possess a 6-, 51-, and 95-fold enhanced *NSP2* expression in root tissue compared to empty vector control plants. First, the root systems of 34-day-old plantlets grown *in vitro* on EKM medium containing low levels of ammonium nitrate (0.375 mM) and high levels of phosphate (3 mM) were analyzed. Control plants transformed with an empty vector showed a highly branched root system with up to 80 secondary lateral roots. This contrasts with what was observed in m **NSP2*ox* lines 3 and 6, in which the root system was less branched, with only ~30 secondary lateral roots, whereas the primary root length is unaffected (**Figures 1B, C**). Such root phenotype was not observed on m **NSP2*ox* line 1. As this line has only moderately levels of transgene expression compared to lines 3 and 6, it suggests that repression of lateral root growth has a threshold of *Pan*NSP2** expression. Next, we analyzed the shoot architecture of 60-day-old plantlets grown on potting soil. In the case of shoot branching, control plants transformed with an empty vector exhibited an average of 11 branches. In contrast, *m*NSP2*ox* lines showed increased shoot branching, with an average of 14 to 16 branches, respectively (**Figure 1D**). This increased number of lateral branches is associated with an increased number of internodes (**Figure 1E**). This phenotype is also observed in m*NSP2*ox line 1, indicating that *NSP2*-controlled developmental effects on plant architecture in root and shoot might have a different threshold. Quantification of internode diameter suggests that ectopic *Pan*NSP2** expression may also increase stem thickness, even though the trees were all similar in size (**Supplementary Figure S3**). Taken together, the data show that ectopic m*NSP2* expression affects shoot and root development in *Parasponia.*

### *Parasponia *NSP2** ox lines possess enhanced mycorrhizal infection

**Figure 2:**
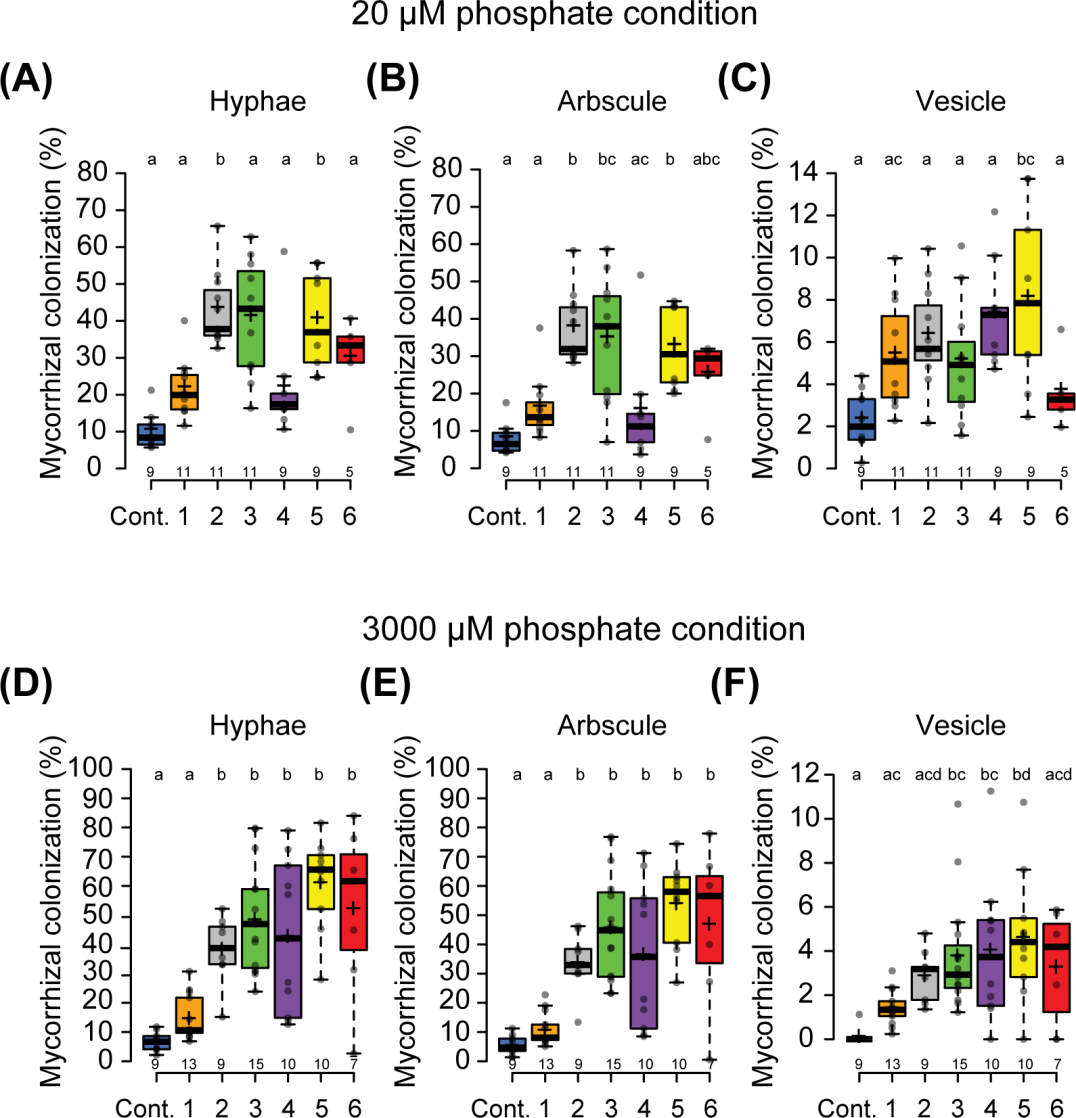
Ectopic expression of *PanNSP2* in *Parasponia* enhances mycorrhizal colonization. Mycorrhizal colonization grown at low exogenous phosphate condition (20 µM PO_4_^3-^) **(A–C)** and high exogenous phosphate condition (3000 µM PO_4_^3-^) **(D–F)**. *Parasponia* lines 1 to 3 are transformed with *pLjUBQ1:mPanNSP2intron* construct, whereas lines 4 to 6 *NSP2* contain *pLjUBQ1:mPanNSP2*. Different letters indicate significant differences (p<0.05) between these lines as determined by One-way ANOVA followed by a Tukey’s *post hoc* test. Plants were harvested 6 weeks post-inoculation (6 wpi) with 300 spores/plant. All data are displayed in box plots, showing data points, the median, and the interquartile range (IQR).

Over-expression of an **NSP2* miR171h* resistant allele in *M. truncatula* roots enhances arbuscular mycorrhizal colonization under phosphate-limited conditions (Lauressergues et al., 2012). Furthermore, a more recent study showed that in *M. truncatula* and barley *miR171h* resistant *NSP2* over-expression promotes arbuscular mycorrhizal root colonization even under exogenous phosphate conditions that are less amenable for the mycorrhizal symbiosis. This response is associated with the up-regulation of the strigolactone biosynthesis pathway (Li et al., 2022). We questioned whether *Parasponia* m **NSP2*ox* lines also possess enhanced mycorrhization colonization levels. We tested two phosphate conditions; 20 μM and 3 mM exogenous Pi, and inoculated all six m **NSP2*ox* lines with *Rhizophagus irregularis* DOAM197198 spores. Mycorrhization levels were scored 6 weeks post inoculation. Empty vector control plants can be colonized under both conditions, though the high exogenous phosphate concentration affects arbuscular mycorrhizal infection levels, most notably the number of vesicles formed. In the m **NSP2*ox* lines, the number of hyphae, arbuscules, and vesicles was significantly increased under both phosphate conditions when compared to the empty vector control line (**Figure 2**). At high exogenous phosphate conditions, the level of mycorrhizal colonization is associated with the level of *Pan*NSP2** expression. Thus, we conclude that ectopic expression of *Pan*NSP2** in *Parasponia* promotes mycorrhization, which is consistent with reported data in *M. truncatula* and barley.

### *NSP2* enhances the expression of genes in the carotenoid biosynthetic pathway under nutrient starved and nodulation permissive growth conditions

**Figure 3:**
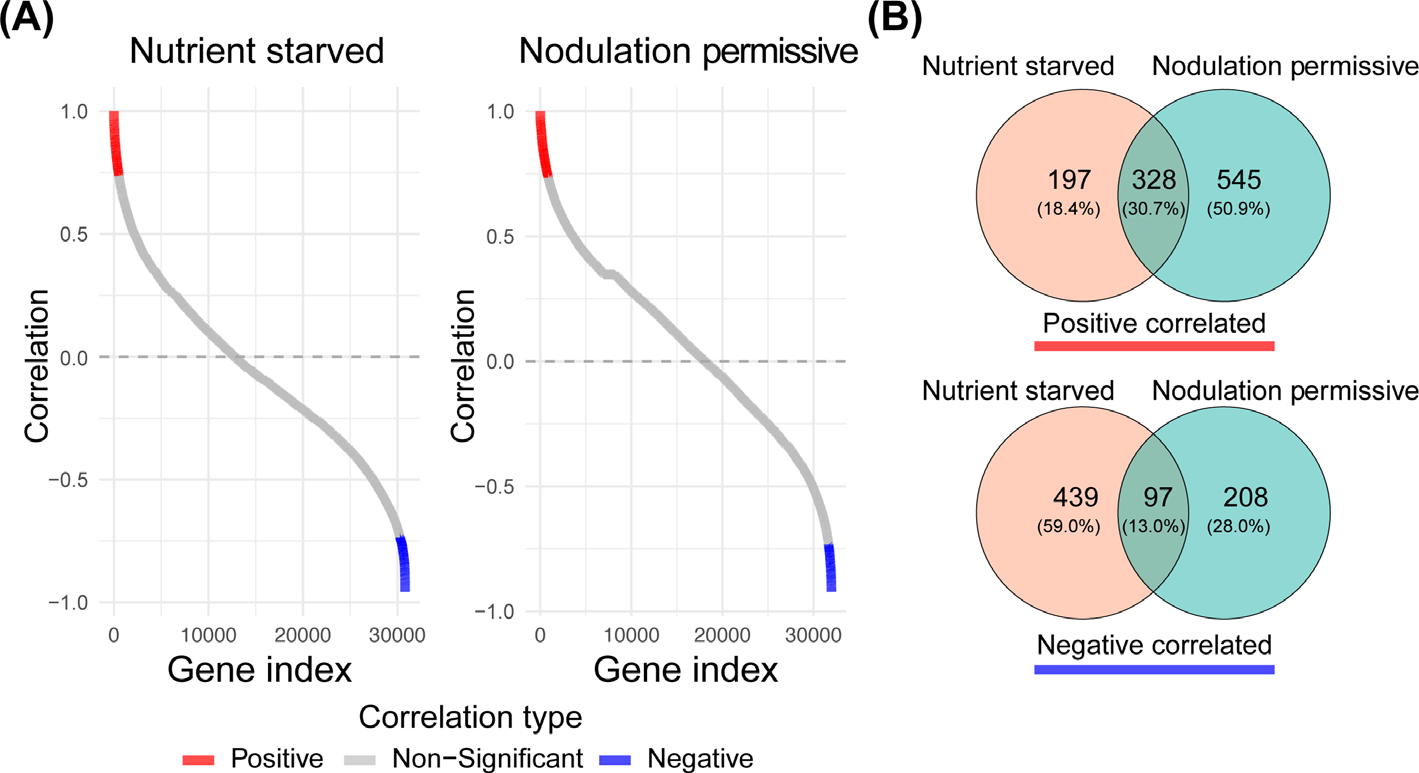
Pearson correlation of all *Parasponia* genes versus *NSP2* profiles under arbuscular mycorrhizal and nodulation permissive nutrient conditions. **(A)** Pearson correlation coefficients are sorted in ascending order. Genes that are significantly positively correlated are colored in red, while negatively correlated genes are colored in blue. (Benjamini-Hochberg adjusted p-value, BH < 0.05) **(B)** Venn diagrams illustrate the overlap between genes that positively and negatively correlate with *NSP2* expression for the two nutrient conditions.

To identify genes that are potentially directly or indirectly regulated by *NSP2* in *Parasponia*, we investigated the transcriptional effect of increased m*NSP2* expression under two distinct nutrient conditions. First plants were grown for 6 weeks on nutrient-rich medium containing 24.72 mM NO₃⁻, 2.6 mM NH₄^+^, and 2.6 mM PO_4_^3-^ and subsequently transferred for 3 weeks to nutrient starved medium (no nitrogen (N) or phosphate (P) source). This treatment will enhance the mycorrhizal responsiveness of the plants. In the second treatment, the plants were transferred from the nutrient-rich medium to nodulation medium, which contains high phosphate levels (0.375 mM NH_4_NO_3_ and 3 mM PO_4_^3-^) (see Materials & Methods). The Pearson correlation test revealed that under nodulation permissive conditions, 2.73% (873 genes) exhibited a positive correlation with *NSP2* expression, while only 0.95% (305 genes) showed a negative correlation (p-value, BH < 0.05). Conversely, under nutrient starved conditions, 1.70% (525 genes) were positively correlated with *NSP2*, and 1.74% (536 genes) were negatively correlated (**Supplementary Table S1**). Most genes displayed a non-significant correlation with *NSP2* under either condition (**Figure 3A**).

We performed a hypergeometric test on the overlap between the positive and negative correlated gene sets under both nutrient conditions. The results indicated a p-value of 0 for the overlap in positively correlated genes and a p-value of 1.250571e^-96^ for the negatively correlated gene set. These findings led us to conclude that *NSP2* consistently regulates a core set of genes, regardless of nutrient conditions, with a subset of genes showing differential regulation attributable to the nutrient environment (**Figure 3B**).

**Figure 4:**
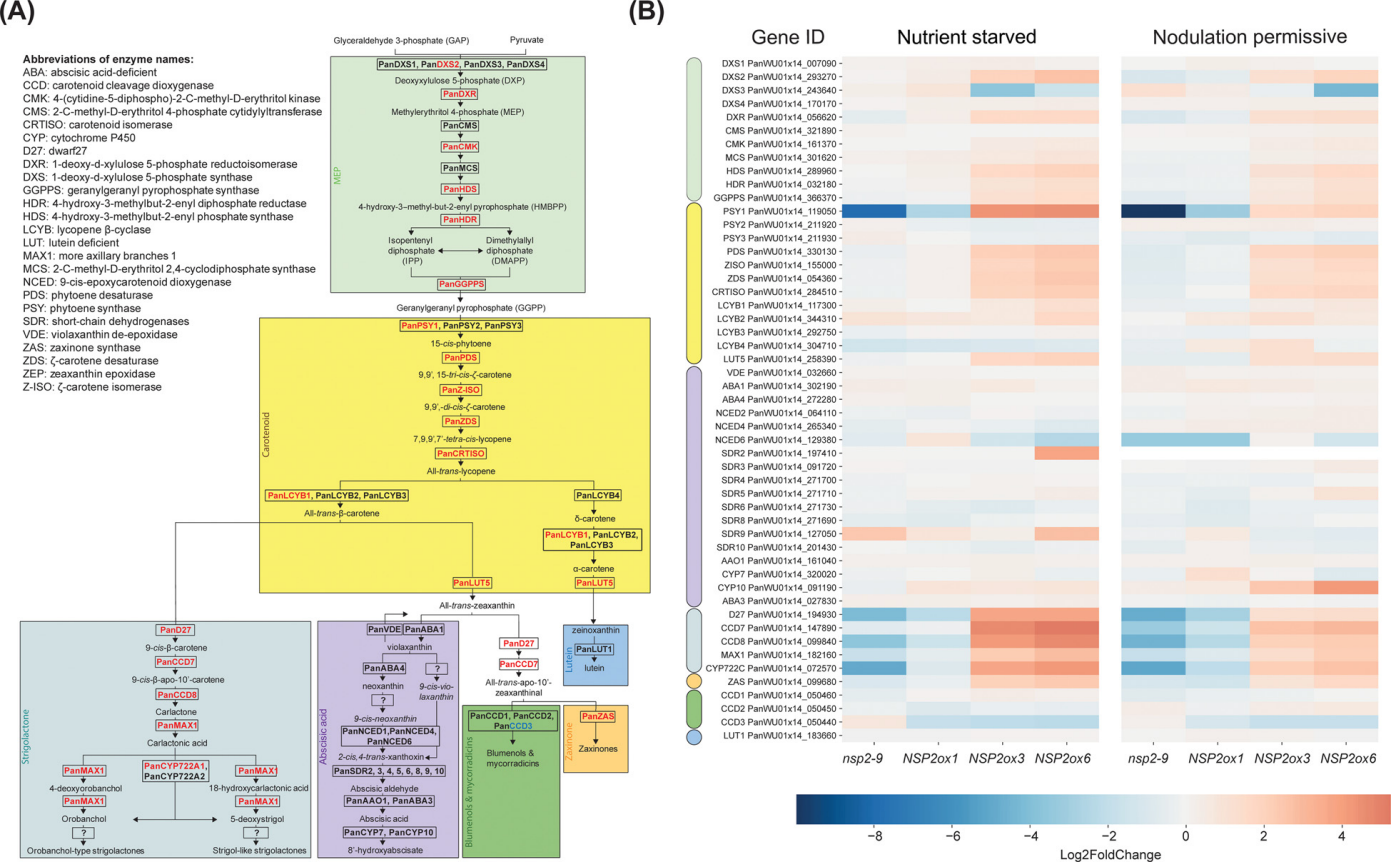
Schematic representation of the carotenoid biosynthetic pathway in *Parasponia* and heatmap of differential gene expression in root tissue in *Parasponia* under nutrient starved mycorrhiza and nodulation permissive conditions. **(A)** *Parasponia* genes found to be positively correlated with *NSP2* over-expression level are colored in red, while non correlating genes are colored in black. The MEP biosynthetic pathway is shaded with green, the carotenoid biosynthetic pathway is shaded with yellow, the strigolactones biosynthetic pathway is shaded cyan, the ABA biosynthesis pathway is shaded with magenta, the zaxinone biosynthesis is shaded orange, the blumenol and Mycorradicins biosynthesis is shaded green, and the Lutein biosynthesis is shaded light blue. Enzyme names are based on annotations in *Arabidopsis thaliana* and *Parasponia andersonii*. Biosynthetic pathway is based on Wakabayashi et al., 2019; Wakabayashi et al., 2020; and Li et al., 2022. **(B)** Heatmap of gene expression in the carotenoid pathway across different lines: *Pannsp2*-9 knockout mutant, m*NSP2*ox1, m*NSP2*ox3, and m*NSP2*ox6 ectopic expression lines. Expression levels are indicated by color intensity, with the scale representing log_2_fold changes. Each row represents a gene from the MEP (green), carotenoid (yellow), ABA (magenta), strigolactone (cyan), zaxinone (orange), blumenols (green, and lutein (blue).

Studies in *M. truncatula*, rice, and barley suggest that ectopic expression of *NSP2* enhances the colonization of plant roots by arbuscular mycorrhizal fungi, potentially through the modulation of the strigolactone biosynthetic pathway (Liu et al., 2011; Li et al., 2022). We investigated the activation of the methylerythritol phosphate (MEP), carotenoid, strigolactone, zaxinone, and abscisic acid (ABA) biosynthetic pathways in roots of *Parasponia* m **NSP2*ox* lines (**Figure 4A**; **Supplementary Table S1**). This revealed a positive correlation between the expression of *NSP2* and all genes encoding enzymes in the carotenoid, strigolactone, and zaxinone pathways (**Figure 4B**; **Supplementary Table S1**). The encoded enzymes of these genes convert geranylgeranyl pyrophosphate at the start of the carotenoid pathway into strigolactones (Okada et al., 2000; Ablazov et al., 2023). In addition, the expression levels of genes required for the biosynthesis of the apocarotenoid zaxinone correlate with the *NSP2* expression. Zaxinone may act in a positive feedforward loop towards strigolactone biosynthesis, as was shown in rice (Votta et al., 2022). Taken together it is most probable that *NSP2* ectopic expression leads to an increased biosynthesis of strigolactones in *Parasponia* root tissue.

Next, we questioned whether the transcriptional activation of the strigolactone biosynthetic pathway also occurs in the shoot of m*NSP2ox* lines. To test this, we used real-time polymerase chain reaction (qRT-PCR) to quantify the expression of three Parasponia genes, *DWARF27 (PanD27), CAROTENOID CLEAVAGE DIOXYGENASE7 (PanCCD7)*, and *PanCCD8*, in shoots of lines 1, 3, and 6 under both nutrient conditions (**Supplementary Figure S2**, **Supplementary Table S4**). This revealed that *NSP2* can also activate the expression of these genes in shoot tissue.

Together, these observations lead us to conclude that enhanced *NSP2* expression in *Parasponia* markedly enhances the expression of genes essential for strigolactone biosynthesis and the apocarotenoid zaxinone across varying nutrient conditions.

### *NSP2* enhances the expression of a limited number of symbiosis genes among which is CYCLOPS

**Figure 5:**
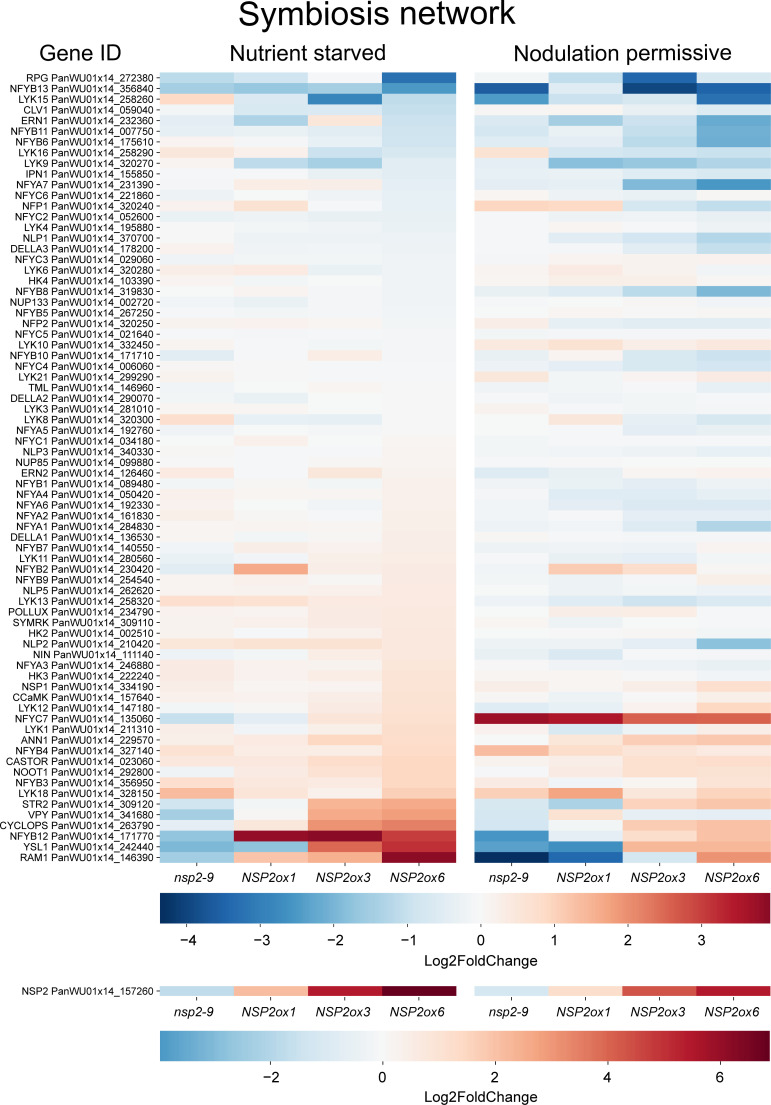
Heatmap of genes involved in the symbiosis network across different lines: *Pannsp2*-9 knockout mutant, m*NSP2*ox1, m*NSP2*ox3, and m*NSP2*ox6 ectopic expression lines. Expression levels are indicated by color intensity, with the scale representing Log_2_fold changes. Each row represents a gene from the symbiosis pathway.

We also analyzed the expression dataset for symbiosis genes of which the expression correlates with m*NSP2* expression. This revealed that the expression of only a limited subset of the symbiosis-related genes positively correlated with m*NSP2* expression (**Figure 5**; **Supplementary Table S1**). Among the positively correlated genes, the transcription factor *PanCYCLOPS*, the two transporters *STUNTED ARBUSCULE2* (*PanSTR2*) and *YELLOW STRIPE-LIKE 1* (*PanYSL1*), and ANNEXIN 1 (*PanANN1)* were shared between both growth conditions. Intriguingly, the genes encoding a nuclear envelope localized cation channel CASTOR and the VAMP-associated protein VAPYRIN (*PanVPY*) show only positive correlation with *NSP2* expression under nodulation permissive conditions, whereas studies in legumes indicated they are also essential for mycorrhizal symbiosis (Imaizumi-Anraku et al., 2005; Pumplin et al., 2010; Murray et al., 2011). Conversely, under nutrient starved conditions, a negative correlation with *NSP2* expression was observed with several transcription factor genes, including *NIN-LIKE PROTEIN1* (*PanNLP1*) and several *NUCLEAR FACTOR Y* genes: *PanNFYA5, PanNFYA6, PanNFYA7, PanNFYB6, PanNFYB8*, and *PanNFYC4* (**Supplementary Table S3**). This indicates a complex regulatory network where enhanced *NSP2* expression differentially influences the expression of a few symbiosis-related genes depending on the nutrient status of the plant. This observation suggests a nuanced role of *NSP2* in the regulatory network governing symbiotic permissiveness.

**Figure 6:**
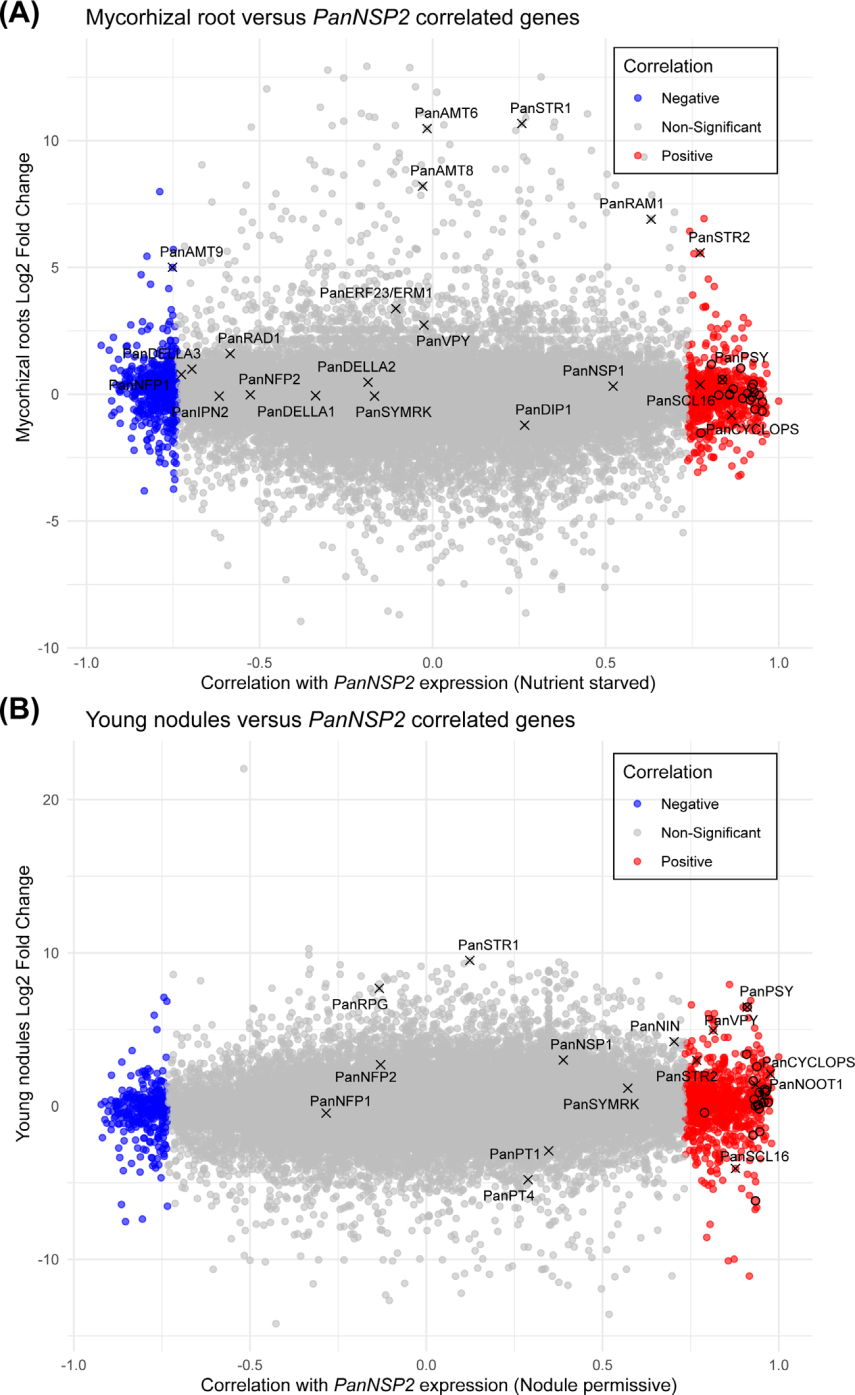
Scatterplot displaying the expression correlation between *mNSP2* and mycorrhizal-induced and nodulation-induced genes. **(A)** Scatterplot representing the Log_2_fold change in gene expression of wild type mycorrhizal *Parasponia* roots against the corresponding Pearson correlation (**Supplementary Table S1**) with *mNSP2* overexpression under nutrient starved conditions. **(B)** Scatterplot representing the Log_2_fold change of gene expression in young wild type *Parasponia* nodules against the corresponding Pearson correlation (**Supplementary Table S1**) with *mNSP2* expression in nodulation permissive conditions. Each point indicates a *Parasponia* gene; blue points denote genes negatively correlated with *mNSP2* expression, red points represent genes with a positive correlation, and gray points signify non-significant correlations. Several marker genes of mycorrhization and/or nodulation are indicated. Genes representing the carotenoid pathway are indicated by a circle.

To investigate the gene expression patterns during mycorrhization and nodulation in *Parasponia* and their interplay with *NSP2* expression, we plotted the Log_2_fold changes of genes in mycorrhizal roots and young developing nodules (**Supplementary Table S3**) against their Pearson correlation coefficients with *Pan*NSP2** expression under both conditions (**Figure 6**). This revealed that *PanSTR2* not only exhibits upregulation in mycorrhizal roots, but also is transcriptionally induced young developing *Parasponia* nodules. When focusing on the carotenoid and strigolactone biosynthetic pathways, only a single gene stood out; namely *PHYTOENE SYNTHASE1* (*PanPSY1*). *Parasponia* possesses three gene copies encoding phytoene synthases (**Supplementary Figure S4**), of which only one correlates with *NSP2* expression. *PanPSY1* is transcriptionally enhanced in mycorrhizal roots (0.58 Log_2_fold (1.5 fold) upregulated) and young nodules (6.47 Log_2_fold (89.7 fold) upregulated) (**Supplementary Table S3**). Phytoene synthase catalyzed the first step in carotenoid biosynthesis and is a major rate-limiting enzyme (Zhou et al., 2022). This suggests that *NSP2*-controlled of *PanPSY1* expression represent a critical step in the biosynthesis of downstream products, including strigolactones in *Parasponia* roots that associate with arbuscular mycorrhizal fungi or nitrogen-fixing rhizobia.

### *Parasponia*NSP2** ectopic expression induces epidermal cell proliferation upon rhizobium inoculation

**Figure 7:**
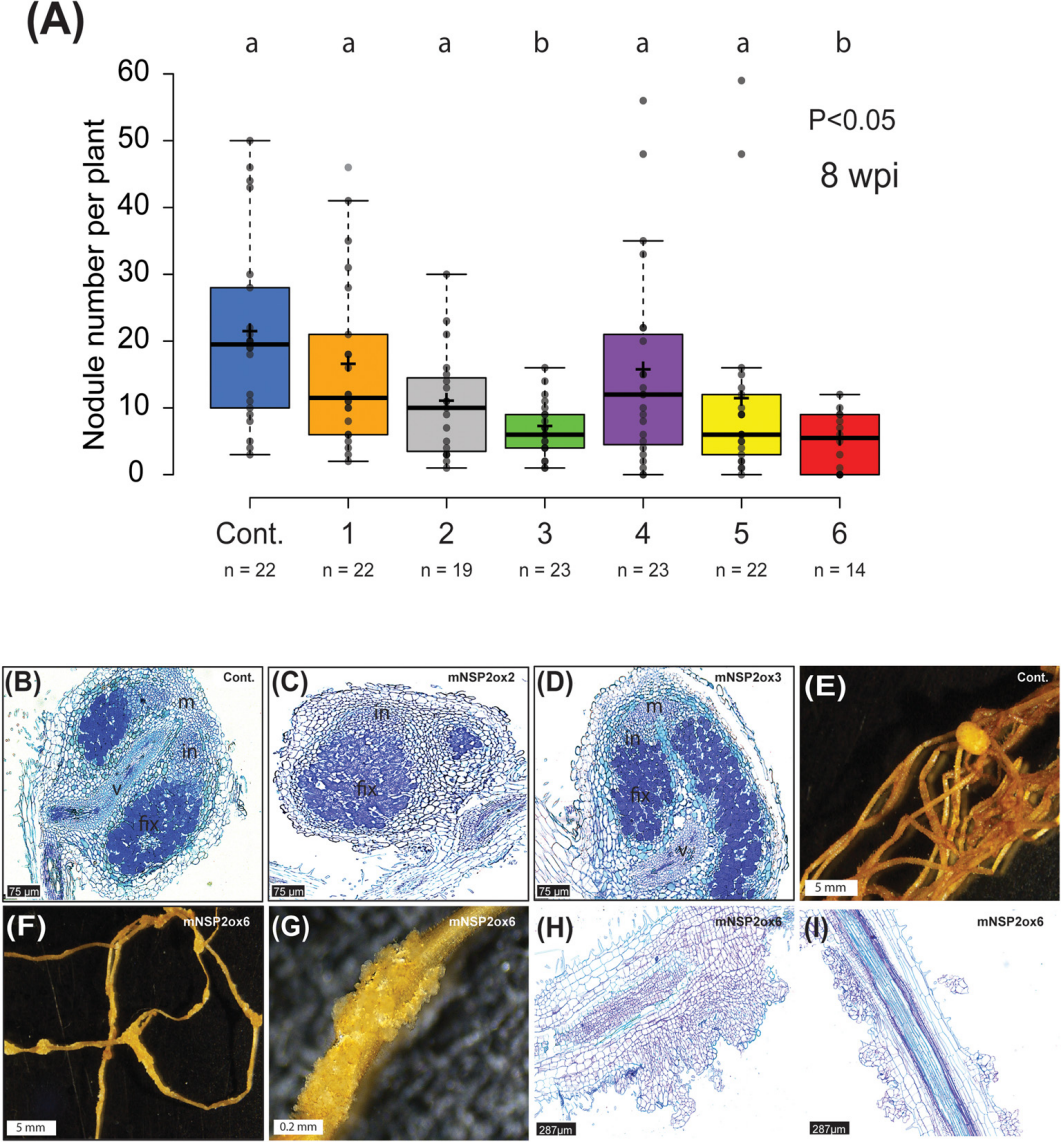
Rhizobium-induced nodulation on *Parasponia NSP2*ox lines. **(A)** Nodule number per plant of empty vector control (Cont.) and mNSP2ox lines 1 to 6. 8 weeks post-inoculation (wpi) with *M. plurifarium* BOR2. Different letters indicate significant differences (p<0.05) between these lines as determined by One-way ANOVA followed by a Tukey’s *post hoc* test. *post hoc* Data is displayed in a box plot, showing data points, the median, and the interquartile range (IQR) **(B–D)** Representative image of a section through a mature nodule formed on an empty vector control line (cont.) **(B)**, mNSP2ox lines 2 **(C)**, and mNSP2ox lines 3 **(D)** (8 wpi). Scale bar is 75 µm. m, nodule meristem; in, infection zone; fix, fixation zone; v, nodule vasculature **(E–G)** Bright-field image of nodulated root of the empty vector control line (cont.) **(E)**, and the root of mNSP2ox line 6 showing rhizobium-induced irregular cell divisions **(F, G)** (10 wpi). Scale bar in **(E, F)** is 5 mm and in **(G)** is 0.2 mm. **(H, I)** Longitudinal sections of rhizobium-induced irregular cell divisions on the root of mNSP2ox line 6 (10 wpi). Scale bar is 287µm.

Given that *NSP2* is critical for both mycorrhization and nodulation, we investigated whether overexpression of m*NSP2* could enhance symbiotic interactions with rhizobia. To test that, we grew all six m*NSP2*ox lines on nodulation medium (0.375 mM NH_4_NO_3_, 3 mM PO_4_^3-^) and scored the number of nodules at 8 weeks post inoculation with *Mesorhizobium plurifarium* BOR2. We observed fewer nodules on lines with the highest m*NSP2* expression (**Figure 7A**). We examined the cytoarchitecture of these nodules, revealing a wild-type phenotype where infection threads and fixation threads were formed (**Figures 7B–D**). A surprising observation was the occurrence of abnormal cell divisions on roots of plants highly expressing m*NSP2*. To determine if this cell proliferation was spontaneous due to the level of *Pan*NSP2** expression or a response to rhizobium application, we grew plantlets of three *Pan*NSP2** over-expressor lines with fold change in gene expression of 6, 51, and 95 on nodulation medium either non-inoculated or inoculated with *M. plurifarium* BOR2. Under the non-inoculated condition, all m **NSP2*ox* lines showed no such epidermal cell divisions, whereas under inoculated conditions, m **NSP2*ox* lines with 51 and 95-fold increase in *m*NSP2** expression, but not 6-fold, exhibited foci of massive cell divisions (**Figures 7E–G**). This shows that high levels of *NSP2* promote cell proliferation in response to rhizobium inoculation. Sections of these aberrant structures revealed mitotic activity in epidermal, cortical and endodermal cell layers, (**Figures 7H, I**). We concluded that increased *NSP2* expression in *Parasponia* negatively regulates nodule formation and causes abnormal proliferation of root tissue in response to rhizobium inoculation.

## Discussion

The GRAS-type transcriptional regulator *NSP2* serves as a conserved hub that regulates the expression of symbiotic genes and genes essential for strigolactone biosynthesis. As such, *NSP2* performs a dual function. The gene is essential for rhizobium-induced root nodule formation in legumes and the Cannabaceae species *Parasponia* (Kaló et al., 2005; Heckmann et al., 2006; van Zeijl et al., 2018; Peng et al., 2021). Additionally, *NSP2*-controlled strigolactones are exuded into the rhizosphere, serving as stimulants for arbuscular mycorrhizal fungi (Liu et al., 2011). Remarkably, ectopic expression of *NSP2* in barley and rice has been shown to increase arbuscular mycorrhizal colonization rates under various nutrient conditions, while the reported trade-offs in plant developmental traits are relatively minor (Li et al., 2022; Yuan et al., 2023). This opens up opportunities to investigate the effects of enhanced *NSP2* expression on plant development and endosymbioses in different species (Isidra-Arellano et al., 2024). We focused on *Parasponia*, the only non-legume known to form nitrogen-fixing root nodules with rhizobium and revealed novel phenotypes associated with increased *NSP2* expression.We generated six independent *Parasponia *NSP2* ectopic* expression lines that differ in *NSP2* over-expression from 6 to 95-fold compared to the root tissue of control plants. These transgenic lines allowed us to correlate phenotypic responses to *NSP2* expression levels. This revealed that the mycorrhizal infection levels positively correlate with *NSP2* expression.

We also explored the ectopic expression lines to identify genes of which the expression correlates positively with *NSP2* expression. Thereby we focused on two gene sets; the pathways that lead to strigolactone biosynthesis and a set of (putative) symbiosis genes. In the case of symbiosis genes, only a few genes showed a correlation with *NSP2* expression (**Supplementary Table S1**). Of these *PanCYCLOPS* is most relevant. CYCLOPS is a transcription factor that functions in the common symbiosis signaling pathway as the last shared hub of arbuscular mycorrhizal- and rhizobium-induced signaling (Capoen and Oldroyd, 2008). Studies in a range of plant species showed that CYCLOPS is critical for both symbioses (Chen et al., 2008; Yano et al., 2008; Horváth et al., 2011; Das et al., 2019; Jin et al., 2018; Prihatna et al., 2018). CYCLOPS and *NSP2* can form a complex having DELLA as an intermediate (Jin et al., 2016). Studies in *M. truncatula* showed that the CYCLOPS orthologs INTERACTING PROTEIN OF DMI3 (*MtIPD3)* and IPD3-LIKE (*MtIPD3L)* are critical for mycorrhizal-induced signaling when plants are grown at higher exogenous phosphate levels (Lindsay et al., 2019). Furthermore, constitutive expression of auto active variants of *MtIPD3* and *MtIPD3L* induces the expression of several arbuscular mycorrhizal symbiosis genes, including *MtVPY* (Lindsay et al., 2019; Lindsay et al., 2022). We found *PanVPY* to be associated with m **NSP2*ox* expression in *Parasponia* when grown at nodulation permissive conditions, which contains a relatively high phosphate concentration, but not under nutrient starved (mycorrhizal permissive) conditions. This led us to conclude that *NSP2*-controlled *PanVPY* expression is phosphate-dependent in *Parasponia*, a response that may require CYCLOPS-DELLA-*NSP2* complex formation.

Analyzing the strigolactone biosynthetic pathways revealed that the expression of all genes encoding essential enzymes for the biosynthesis of carotenoids, strigolactones, and zaxinone positively correlates with *NSP2* expression. In contrast, side branches leading to abscisic acid and lutein do not show such a response. A critical gene in the carotenoid pathway, namely *PanPSY1*, is also enhanced upon rhizobium and mycorrhizal inoculation. PSY commits the first step in the carotenoid pathway converting geranylgeranyl pyrophosphate into 15-cis-phytoene and is generally considered the rate-limiting step in carotenoid biosynthesis (Zhou et al., 2022). PSY functioning is controlled by several mechanisms, in which transcriptional regulation plays an important role (Stanley and Yuan, 2019). Enhancing *PSY* expression has been utilized extensively in plant biotech approaches to enhance carotenoid biosynthesis (Zhou et al., 2022). We hypothesize that in *Parasponia* the symbiotic regulation of *PanPSY1* in response to arbuscular mycorrhizal infection or rhizobium-induced nodulation is a critical factor ultimately in the biosynthesis of strigolactones.

High expression levels of *NSP2* also cause pleiotropic phenotypes. Some of these, like reduced axillary bud outgrowth, increased secondary thickening of the stem, and reduced lateral root formation are known strigolactone -controlled responses (Waters et al., 2017). In addition, we observed irregular cell divisions upon rhizobium-inoculation in roots of some *Parasponia* m **NSP2*ox* lines. The structures formed resemble enlarged pre-nodules, which can serve as infection pockets to facilitate rhizobial crack entry (Lancelle and Torrey, 1984). A function of *NSP2* in the formation of rhizobium infection pockets is also known in the legume *Aeschynomene avenia*. Like in *Parasponia*, rhizobium penetrates *A. avenia* by crack entry. Under nodulation permissive conditions, but in absence of rhizobium, *A. avenia* forms clusters of multicellular auxiliary hair-like cells at the base of newly formed lateral roots. These structures can be explored by rhizobium as an infection pocket that guides bacterial entry (Bonaldi et al., 2011). *A. avenia nsp2* mutants do not form such infection pockets (Quilbé et al., 2022). Together, these experiments suggest the importance of a balanced *NSP2* expression in the formation of pockets that ultimately can be explored by rhizobium as a starting point of intracellular infection.

Ectopic expression of *NSP2* also affects plant architecture. m **NSP2*ox Parasponia* trees have a reduced number of lateral roots but more lateral shoot branches. Studies in rice revealed similar developmental phenotypes, where *Os*NSP2*ox* results in increased tiller formation and a reduced number of lateral roots (Liu et al., 2011; Yuan et al., 2023). These phenotypes question whether exploring *NSP2* ectopic expression in a biotech approach aiming to improve crop yield in low phosphate environments is a realistic strategy (Isidra-Arellano et al., 2024). Our findings advocate using tissue-specific promoters to obtain enhanced mycorrhization while minimizing pleiotropic phenotypes in plant architecture. Studies in rice indicate that using native promoter elements to enhance expression of GRAS-type transcriptional regulators might be the way forward to improve agronomic traits under low and medium-phosphorus conditions (Yuan et al., 2023).

## Materials and methods

### Plant materials and growth conditions

The tissue culture propagation and maintenance of *Parasponia* plants were done on agar plates supplemented with Parasponia propagation medium. This medium contains 3.2 g/L SH-basal salt, 1 g/L SH-vitamin mixture, 10 g/L sucrose, 1 mL/L BAP (1 mg/mL), 100 μL/L IBA (1 mg/mL), 3 mL/L MES (1 M, pH 5.8), and 8 g/L Daishin agar (van Zeijl et al., 2018; Wardhani et al., 2019). Performance of nodulation and mycorrhization assays was carried out on platelets vegetatively grown and rooted following previously published procedures (van Zeijl et al., 2018; Wardhani et al., 2019).

### Nodulation assay and analysis

Rooted plantlets were grown in 1 liter crystal-clear polypropylene pots (OS14BOX, Duchefa Biochemie, Netherlands) half-full, with a mixture (1:2 weight ratio) of river sand and agri perlite (Massmond-Westland, Netherlands). The mixture was watered with modified EKM medium [15 μM Fe-Citrate, 3 mM MES (pH 6.6), 6.6 μM MnSO_4_, 4.1 μM Na_2_MoO_4_, 2.08 mM MgSO_4_, 0.70 mM Na_2_SO_4_, 1.4 mM CaCl_2_, 0.375 mM NH_4_NO_3_, 1.5 μM ZnSO_4_, 0.88 mM KH_2_PO_4_, 1.6 μM CuSO_4_, 2.07 mM K_2_HPO_4_, and 4 μM H_3_BO_3_] (Becking, 1983) and inoculated with *Mesorhizobium plurifarium* BOR2 (OD600 = 0.025). Inoculated plants were then incubated in a controlled climate room at 85% humidity, under a 16/8 h day/night growth condition for eight weeks. Plants were removed from pots and washed under running tap water followed by quantification of total nodule number and harvest of some nodules for sectioning. Subsequently, harvested nodules were fixed in 0.1 M phosphate buffer (pH 7.2) containing 0.5% glutaraldehyde followed by vacuum application for 60 minutes. Next, nodules were embedded in infiltration plastic, Technovit 7100 (Heraeus-Kulzer, Germany), following the protocol provided by the manufacturer. A Leica RJ2035 microtome was used to make 4.5 μm plastic sections which were stained with 0.5% toluidine blue. A Leica DFC425c camera was used to make high resolution images of the cytoarchitecture of the fixed nodules.

### Mycorrhization assays and ink staining

Rooted plantlets were placed in pots half-filled with a mixture (5:1 weight ration) of river sand and commercial potting soil and watered with half-strength Hoagland’s medium containing 20 μM phosphate (low P condition) or 3 mM (high P condition). Plants then were incubated in a controlled climate room at 85% humidity, under a 16/8 h day/night regime for two weeks followed by inoculation with 300 spores of *Rhizophagus irregularis* fungi (Agronutrion-DAOM197198, Carbonne, France) and subsequent incubation for 6 weeks. Afterwards, plants’ roots were harvested and treated with 10% (w/v) KOH and boiled for 30 minutes at 90°C and then stained in a staining solution contains 5% acetic acid and 3% ink (Waterman Vulpen, Zwolle, the Netherland) for 15 minutes at 90 °C. Next, fungal colonization and formation of arbuscules and vesicles were quantified, using the gridline intersect method (Giovannetti and Mosse, 1980), and high-resolution images were taken, using a Leica CTR66000 microscope.

### Vector constructs

All vectors used in this study were propagated in *Escherichia coli* strain DH5α and assembled, using Golden Gate Cloning (GGC) as was previously described (Engler et al., 2009). Level one and two acceptors and binary vectors used for the assembly were obtained from the GGC toolbox (Engler et al., 2014). *Parasponia* clone of *NSP2* coding sequence (CDS) with and without insertion of intron 10 of *Arabidopsis thaliana UBQ10* gene (intron) in the first putative splicing site in *NSP2* CDS, including silent mutation in microR171h putative site and golden gate BsaI or BpiI restriction sites was synthesized as Level zero vectors. Subsequently, level zero vectors of *NSP2*, *Lotus japonicus UBQ1* (*LjUBQ1*) promoter, C-terminal Myc tag, and *Agrobacterium tumefaciens* nopaline synthase terminator (T-NOS) were recombined into level one acceptor. Finally, level one vectors of *NSP2* with or without (intron), including Kanamycin resistant Level one vectors were recombined into Level 2 binary vectors. All binary GGC vectors were validated by restriction enzymes and sequencing before transformation.

### Plant transformation and Genotyping

Level 2 binary vectors were transformed into *Agrobacterium tumefaciens* AGL1 strain, using Eppendorf Eporator (Eppendorf SE, Hamburg Germany). Transformed AGL1 were then grown on *lysogeny broth* (LB) agar plate supplemented with appropriate antibiotics and incubated at 28°C for 2 days. *Parasponia* plant transformation was carried out as previously described (van Zeijl et al., 2018; Wardhani et al., 2019), so young stems and petioles were collected from *Parasponia* trees grown in controlled greenhouse conditions at 85% humidity and under a 16/8 h day/night regime. The surface of the stems and petioles was sterilized with 2.5% bleach with a few drops of Tween 20, and subsequently stems and petioles washed six times with sterile water followed by a co-cultivation with AGL1 strains carrying constructs of interest for 2 days. Afterwards, transformed stems and petioles were transferred to callus induction medium supplemented with cefotaxime (300μg/ml) and kanamycin (50μg/ml) antibiotics for one week followed by transferring to plant propagation medium supplemented with cefotaxime (300μg/ml) and kanamycin (50μg/ml) antibiotics, refreshed every 1-2 weeks until transgenic shoots were formed. Genotyping was performed using a pair of primers designed to anneal to the *L. japonicus UBIQUITIN 1* promoter (pLjUBQ1) and the C-terminal Myc tag, with amplification carried out using the Phire Plant Direct PCR kit (Thermo Fisher, F130WH), following the manufacturer’s protocol.

### Plant growth conditions for RNA-sequencing

*Parasponia* plant lines, consisting of three *NSP2* overexpression mutants (mNSP2ox1, mNSP2ox3, and mNSP2ox6), a *nsp2* knockout (*nsp2-9*), and control line 46 with an empty vector were selected. The *NSP2* overexpression mutants displayed *NSP2* expression levels of 6, 51, and 95-fold higher than empty vector control. Selected plants were cultivated for 6 weeks on nutrient rich medium containing 24.72 mM NO₃⁻, 2.6 mM NH₄^+^ and 2.6 mM PO_4_^3-^. Next, cultivated plants were transferred for 3 weeks to either nutrient starved medium (no N and P resource), or the nodulation permissive medium (0.375 mM NH4NO3 and 3 mM PO4^3-^).

Subsequently, healthy plantlets were transferred to pots containing a 2:1 weight ratio of perlite to sand. Each pot was supplemented with 150 mL of EKM medium, with or without nitrogen and phosphorus, referred to as nodulation permissive (low nitrate, high phosphate) and nutrient starved (low nitrate and low phosphate) treatments, respectively. Pots were populated with three plantlets of a single genotype and grown under a 16-hour light/8-hour dark photoperiod for 21 days. Following this period, roots from each pot were pooled for RNA extraction.

### RNA extraction and quality control

For each treatment, five root samples weighing 50 mg each were collected. Tissue lysis was performed using a QIAGEN TissueLyser LT, followed by sequential phenol:chloroform and chloroform extractions. Nucleic acid precipitation was performed using sodium acetate, followed by sequential washing steps with isopropanol and ethanol to purify the samples. Subsequently, the samples were treated with DNase to eliminate genomic DNA contamination. A second round of phenol:chloroform and chloroform extractions was conducted. The RNA was then re-precipitated in ethanol and re-suspended in nuclease-free water. The concentration of the isolated RNA was quantified using both a Biochrom SimpliNano™ Spectrophotometer and a Qubit 2.0 Fluorometer, utilizing Broad Range Assay Kits. RNA integrity was confirmed through gel electrophoresis. To validate *NSP2* expression levels, quantitative polymerase chain reaction (qPCR) was employed. *ACTIN* and *ELONGATION FACTOR1 ALPHA* (*EF1α*) served as the housekeeping genes for normalization.

### RNA-seq library preparation and high-throughput sequencing

For each harvested sample, mRNA was isolated from total RNA utilizing poly-T oligo-attached magnetic beads. The resulting mRNA served as the template for first-strand cDNA synthesis, which was initiated with random hexamer primers, and subsequently followed by second-strand cDNA synthesis. Sample quality and nucleic acid concentration were quantitatively assessed using Qubit and qPCR assays, using *EF1α* and *ACTIN* for normalization genes. Size distribution of the samples was further confirmed via bioanalyzer analysis. Three biological replicates for each sample exhibiting the best characteristics (quantity and quality) for sequencing were selected for library preparation and sequencing, conducted at Novogene UK (Cambridge, United Kingdom). The resulting libraries were pooled and subjected to paired-end sequencing on an Illumina NovaSeq 6000 platform. Each biological replicate generated approximately 25 million reads, with each read consisting of 150 base pairs.

The quality of the sequenced reads was assessed using FastQC and subsequently visually inspected through MultiQC. Remaining adapters and low-quality bases were trimmed using Trimmomatic with settings TRAILING:3, SLIDINGWINDOW:4:15, MINLEN:50, HEADCROP:12. Transcript abundances were quantified with Kallisto, and the resulting files were loaded into R Studio version 2022.07.2 using R version 4.1.1. Differential gene expression analysis was conducted using DESeq2 for each condition (Nodulation permissive and nutrient starved) separately (**Supplementary Table S2**).

### Correlation Analysis between *NSP2* Overexpression and Gene Expression

Pearson correlation analysis was performed for each nutrient condition (nodulation permissive and nutrient starved) to assess the expression correlation with *NSP2*. The analysis was conducted in R, calculating the correlation, p-value, and False Discovery Rate (FDR, or adjusted p-value). An FDR cutoff of 0.05 was applied and used to categorize the correlation as positive, non-significant, or negative. The Pearson correlation graphs were plotted using the ggplot2 package. Overlapping genes between nutrient conditions were visualized using the VennDiagram package in R.

Prominent pathways featuring upregulated genes under *NSP2* overexpression conditions, as well as genes of interest related to nodulation and mycorrhization in *Parasponia*, were selected for detailed investigation. The first set, named the “Symbiosis Network,” consists of a gene set in *Parasponia* previously annotated (**Supplementary Table S1**). The second set includes genes involved in the carotenoid biosynthesis pathway in *Parasponia*. Orthologous genes in *Parasponia* involved in carotenoid biosynthesis were identified using gene IDs from *Arabidopsis thaliana*, as reported in the study by (Li et al., 2022) (**Supplementary Table S1**). Corresponding gene IDs for *Parasponia* were identified through a reciprocal best BLAST hit approach, using the *Arabidopsis* gene IDs as references. BLASTP was performed with the following parameters: E-value cutoff of 1e-5, BLOSUM62 scoring matrix, gap penalties of 11 for existence and 1 for extension, and a word size of 3. The top BLAST hits in *Parasponia* were then used for a reverse search against the TAIR Araport 11 protein set to verify orthologous relationships.

### Data analysis on mycorrhization and Young nodules data

*Parasponia* wild type young nodule data were previously published under PRJNA272473. Additionally, an experiment was conducted on *Parasponia* plants inoculated with *Rhizophagus irregularis* (MYC+) or left uninoculated (MYC-) [(van Velzen et al., 2018; van Zeijl et al., 2018; Bu et al., 2020; Rutten et al., 2020; Alhusayni et al., 2023)]. *Parasponia* plants were grown under controlled greenhouse conditions with a 16-hour light/8-hour dark photoperiod at 28°C and 85% humidity. The growth medium consisted of a mixture (5:1 ratio) of river sand and commercial potting soil and watered with half-strength Hoagland solution containing 20 μM PO₄^3-^ every other week. After placing the plants in pots, they were allowed to recover for 2 weeks before being inoculated with 300 R. irregularis spores. Root samples were harvested 42 days after inoculation.

Total RNA was extracted from collected samples using the RNeasy Plant Mini Kit (Qiagen). mRNA was isolated from total RNA using poly-T oligo-attached magnetic beads. The isolated mRNA served as the template for first-strand cDNA synthesis, initiated with random hexamer primers, followed by second-strand cDNA synthesis. Sample quality and nucleic acid concentration were assessed using Qubit and qPCR assays. Size distribution of the samples was confirmed via bioanalyzer analysis. Three biological replicates per sample exhibiting the best characteristics (quantity and quality) were selected for library preparation and sequencing.

Sequencing was conducted at Novogene UK (Cambridge, United Kingdom) using an Illumina NovaSeq 6000 platform. Each biological replicate generated approximately 25 million paired end reads of 150 base pairs. The quality of the sequenced reads was assessed using FastQC and subsequently visually inspected through MultiQC.

To analyze the RNA-seq dataset from nodule stages 1, 2, and 3 versus roots, as well as mycorrhization data of *Parasponia* wild type MYC- and MYC+ data, the following procedures were performed using the R programming language. Transcript abundances were quantified using Kallisto. The resulting files were imported into R Studio version 2022.07.2 with R version 4.1.1 for subsequent analysis using DESeq2 as previously described (**Supplementary Table S2**). The differential expression results were integrated with existing annotated datasets for nodulation permissive and nutrient starved conditions. Ggplot2 was used to plot Log_2_fold change versus the *NSP2* correlation (**Supplementary Table S3**).

### Statistical analysis

Box plot graphs were generated using BoxPlotR, an online tool developed by (Spitzer et al., 2014). Bar charts were created in Microsoft Excel for Mac (Version 16.89). The Kruskal–Wallis test, followed by Tukey’s *post-hoc* test, was employed for statistical analysis. Statistical significance was established at a threshold of p < 0.05. Additionally, the quantification of internode diameter was assessed using a Student’s t-test, with statistical significance also defined as p < 0.05.

## Data Availability

The RNA-seq datasets analyzed in this study are available in the NCBI SRA repository under BioProject number PRJNA1029573. Plant material and seeds used in this study can be obtained upon request from the authors. Scripts used in the differential gene expression and visualizationvisualisation of the heatmaps can be found on GitHub: https://github.com/kleinjoel/NSP2_overexpression_analysis. Young nodules data were previously published under BioProject number PRJNA272473.

## Supplementary material

Supplementary Figure S1 Construct design to ectopically express *Parasponia *NSP2**. **(A)** Alignment of putative miR171h target sites in *Medicago truncatula* (Lauressergues et al., 2012) and *Parasponia *NSP2** as well as m*NSP2* a modified version of Parasponia *NSP2* in which the target site of miR171hR is silenced. Matching nucleotides at the miR171h binding site are shown in red, mismatching nucleotides in black, and silencing nucleotides in green. **(B)** A schematic representation of the transformation constructs to achieve ectopic expression of *Pan*NSP2** in *Parasponia*.

Supplementary Figure S2 Ectopic *Pan*NSP2** expression enhances the expression of strigolactone biosynthesis genes in shoot tissue. **(A–D)** qRT-PCR-based expression of *Pan*NSP2*, PanCCD7, PanCDD8*, and *PanD27* in shoot of plants grown under nodulation permissive condition (n=3), and **(E–H)** nutrient starved condition (n=3).

Supplementary Figure S3 Shoot phenotype of *Parasponia *NSP2** ox lines. *post hoc* quantification of internode diameter of the empty vector control line (cont.) and *m*NSP2** ox lines 1,3 and 6. Asterisk indicates significant difference relative to cont. (p < 0.05) as determined by Student’s t-test (*p < 0.05). Stem diameters were measured at the middle of internodes (IN) for the first 12 internodes, from the bottom to the top of 60 days old plants (n=10).

Supplementary Figure S4 Phylogenetic relation of *Parasponia PSY* proteins. Phylogenetic relation is reconstructed based on an alignment of protein sequences from the following species: *Parasponia andersonii, Trema orientalis, Arabidopsis thaliana, Medicago truncatula, Glycine max, Fragaria vesca, Eucalyptus grandis*, and *Manihot esculenta. Parasponia* and *Arabidopsis* proteins are in bold letter-type. Three orthogroups are recognized and marked in distinct colors. Node labels indicate posterior probability.

Supplementary Table S1 RNA-seq analysis of m*NSP2* overexpression in *Parasponia*. Pearson correlation tests and a quantitative analysis of gene correlations under mycorrhizal and nodulation permissive conditions. The table also details genes in carotenoid biosynthesis and symbiosis networks, highlighting their correlation values in relation to *Pan*NSP2** overexpression.

Supplementary Table S2 Integrated RNA-seq Analysis of *Pan*NSP2** overexpression in *Parasponia*, Log_2_fold change (LFC) values for all *Parasponia* genes under both nodulation permissive and nutrient starved conditions, detailing results from m*NSP2* overexpressing and knockout lines.

Supplementary Table S3 RNA-seq analysis of *Parasponia* genes under arbuscular mycorrhiza permissive conditions. This table includes base means, Log_2_fold changes between non-mycorrhized and mycorrhized roots, and adjusted p-values (padj). It also provides annotations, Pearson correlation values for *NSP2* under nutrient starved and nodulation permissive conditions, along with their respective p-values, false discovery rates (FDR), and correlation types.

Supplementary Table S4 Primers used in this study. List of primers, their purpose, and their sequence that were used in various applications in this study.
