# Supplementary material for: Ectopic expression of the GRAS-type transcriptional regulator NSP2 in Parasponia triggers contrasting effects on symbioses
Source: Front Plant Sci. 2024 Oct 30;15:1468812. doi: 10.3389/fpls.2024.1468812 (PMC11557437; doi:10.3389/fpls.2024.1468812)

(A)

miR171h binding site	GAGUGAUAUUGAUUCGGCUCG
MtNSP2 - putative miR171h binding site	GAGUGAUAUUGAUCCGGCUCA
PanNSP2 - putative miR171h binding site	GGGUGAUAUUGGUUCGGCUCA
mNSP2 miR171h (silenced)	GAGUAAUCUUAGUCCGCCUAA

(B)

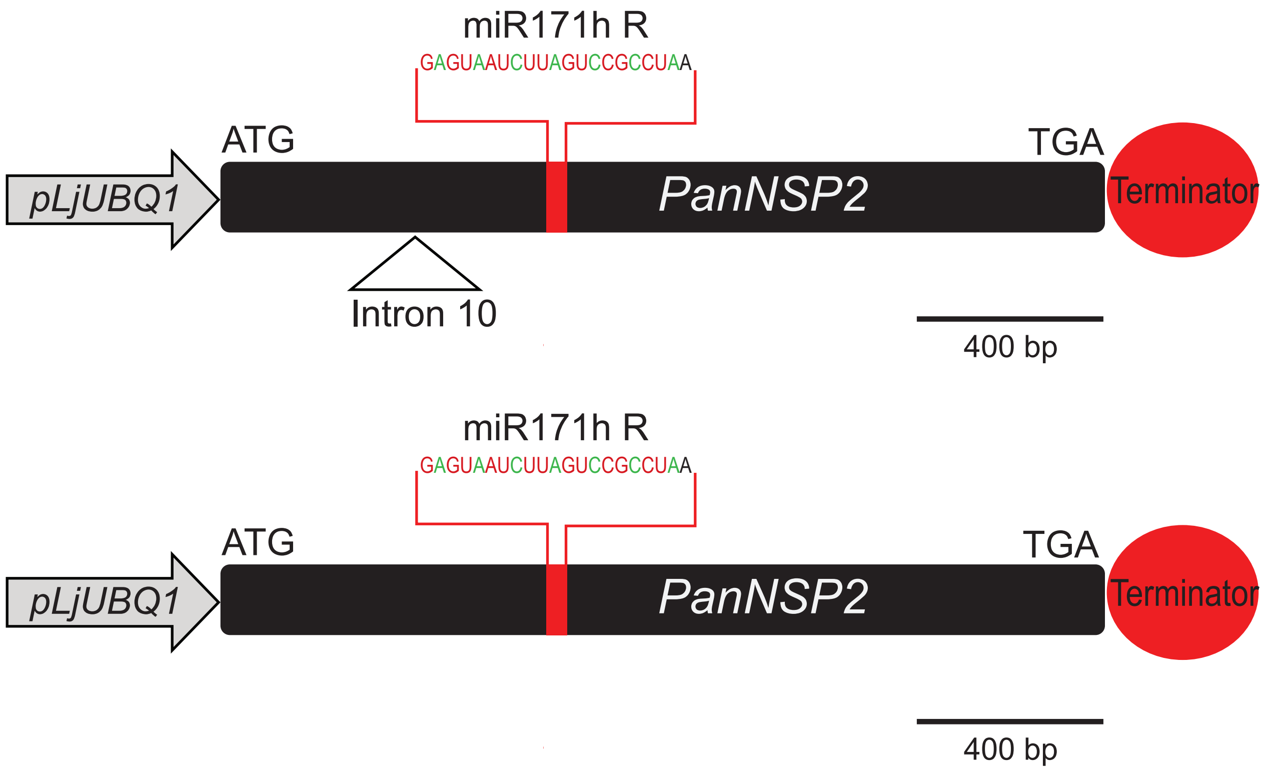

Supplement: Supplementary Figure S1 — Construct design to ectopically express Parasponia NSP2 . (A) Alignment of putative miR171h target sites in Medicago truncatula (Lauressergues et al., 2012) and Parasponia NSP2 as well as mNSP2 a modified version of Parasponia NSP2 in which the target site of miR171hR is silenced. Matching nucleotides at the miR171h binding site are shown in red, mismatching nucleotides in black, and silencing nucleotides in green. (B) A schematic representation of the transformation constructs to achieve ectopic expression of PanNSP2 in Parasponia. [file DataSheet1.pdf]
