# Supplementary figures and images for: Ectopic expression of the GRAS-type transcriptional regulator NSP2 in Parasponia triggers contrasting effects on symbioses
Source: Front Plant Sci. 2024 Oct 30;15:1468812. doi: 10.3389/fpls.2024.1468812 (PMC11557437; doi:10.3389/fpls.2024.1468812)

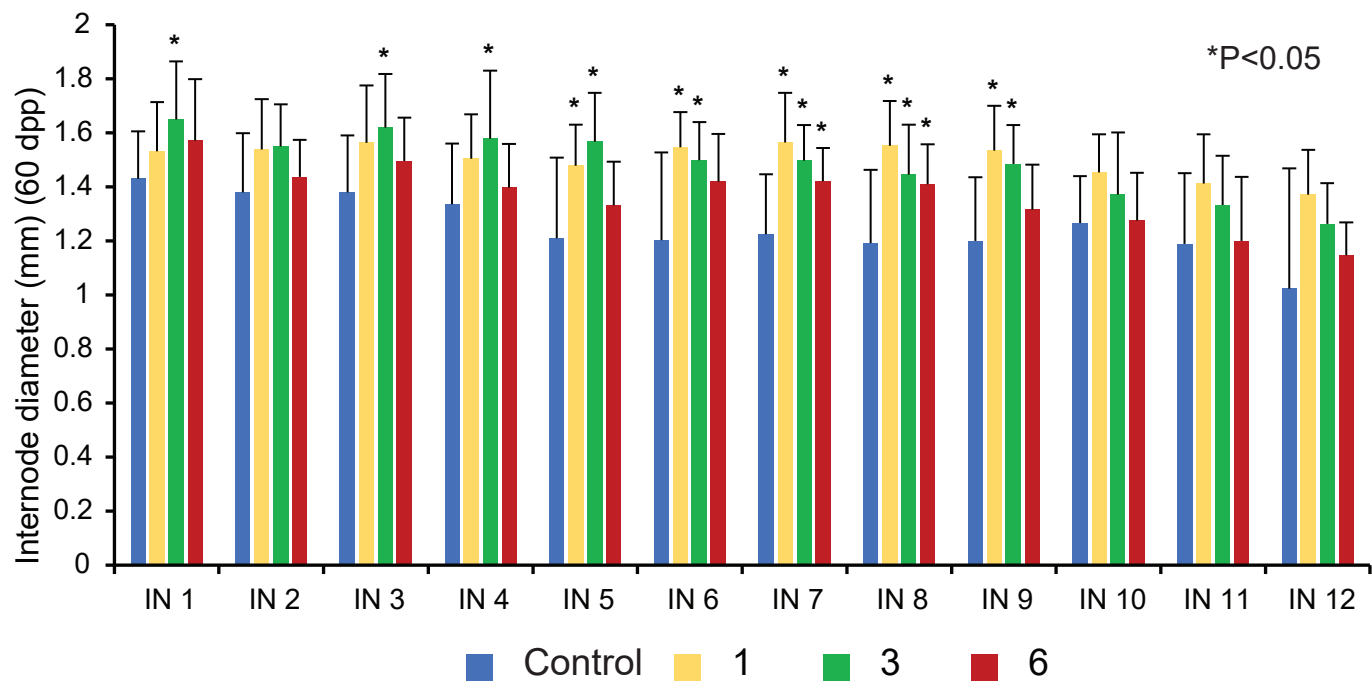

Supplement: Supplementary Figure S2 — Ectopic PanNSP2 expression enhances the expression of strigolactone biosynthesis genes in shoot tissue. (A–D) qRT-PCR-based expression of PanNSP2, PanCCD7, PanCDD8, and PanD27 in shoot of plants grown under nodulation permissive condition (n=3), and (E–H) nutrient starved condition (n=3). [file DataSheet2.pdf]

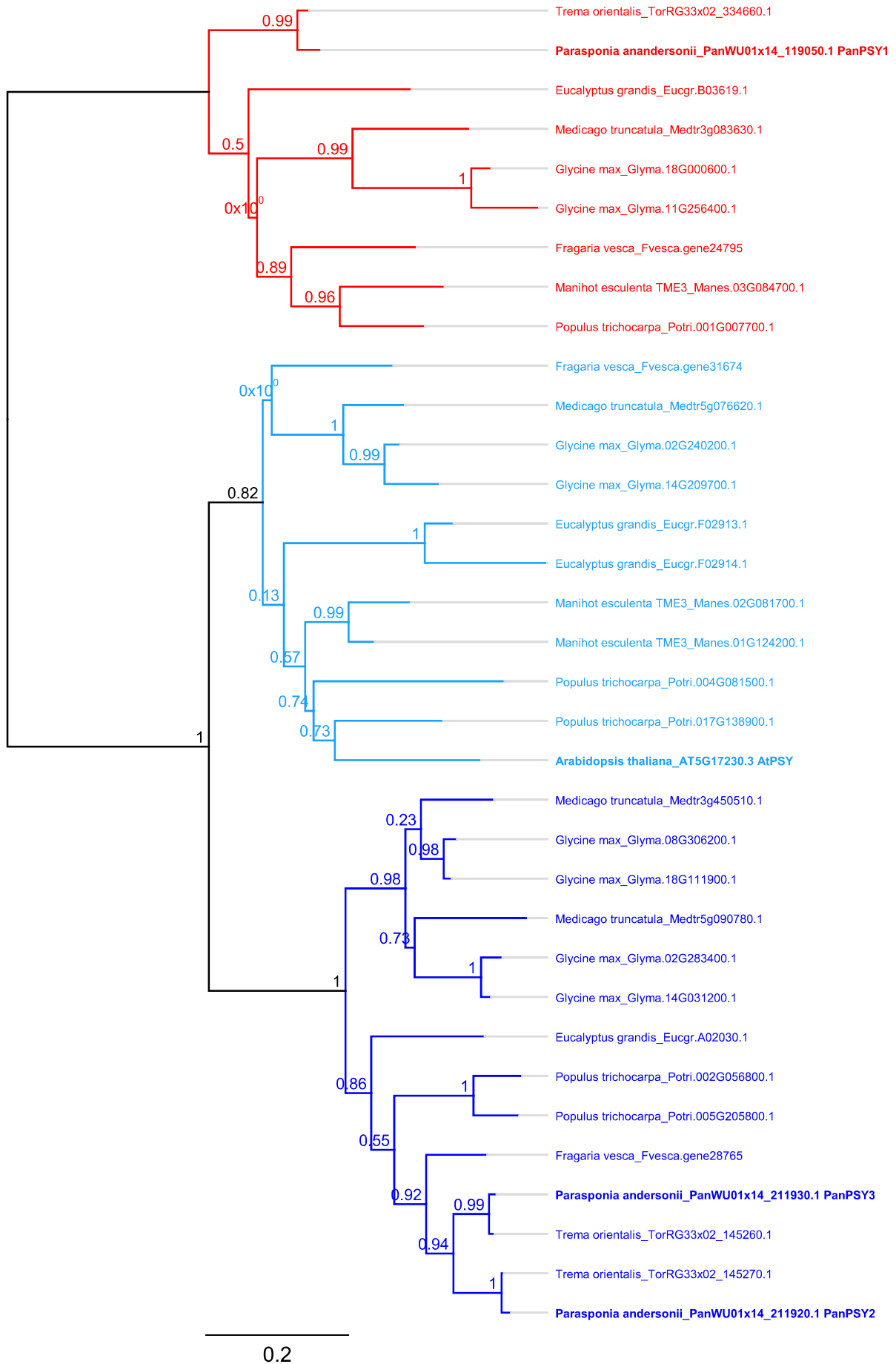

Supplement: Supplementary Figure S3 — Shoot phenotype of Parasponia NSP2 ox lines. post hoc quantification of internode diameter of the empty vector control line (cont.) and mNSP2 ox lines 1,3 and 6. Asterisk indicates significant difference relative to cont. (p < 0.05) as determined by Student’s t-test (*p < 0.05). Stem diameters were measured at the middle of internodes (IN) for the first 12 internodes, from the bottom to the top of 60 days old plants (n=10). [file DataSheet3.pdf]

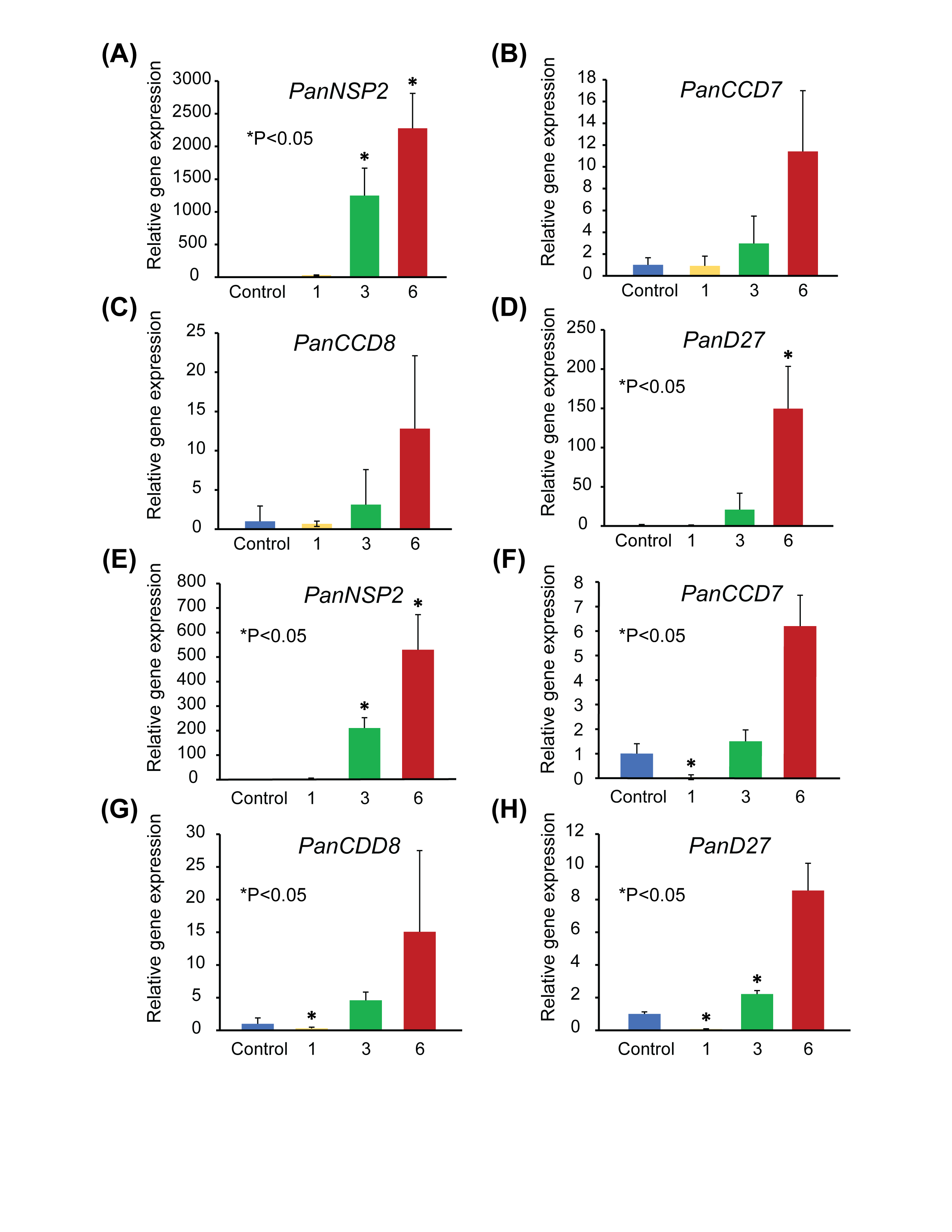

Supplement: Supplementary file 8 [file Image1.tif]
