# Supplementary material for: Ectopic expression of the GRAS-type transcriptional regulator NSP2 in Parasponia triggers contrasting effects on symbioses
Source: Front Plant Sci. 2024 Oct 30;15:1468812. doi: 10.3389/fpls.2024.1468812 (PMC11557437; doi:10.3389/fpls.2024.1468812)
Supplement: Supplementary Figure S4 — Phylogenetic relation of Parasponia PSY proteins. Phylogenetic relation is reconstructed based on an alignment of protein sequences from the following species: Parasponia andersonii, Trema orientalis, Arabidopsis thaliana, Medicago truncatula, Glycine max, Fragaria vesca, Eucalyptus grandis, and Manihot esculenta. Parasponia and Arabidopsis proteins are in bold letter-type. Three orthogroups are recognized and marked in distinct colors. Node labels indicate posterior probability. [file DataSheet4.pdf]

Name	Purpose	Sequence
PanNSP2_For	qRT-PCR	AGCTGTTGCTGGGATTGTTC
PanNSP2_Rev	qRT-PCR	GACTTCCAACCCAAAACGAG
Pan Ef-1a_For	qRT-PCR	AGACAAGGTTAAGCGTGCAG
Pan Ef-1a_Rev	qRT-PCR	TGCAACTGGGCAACAAACTC
PanACT_For	qRT-PCR	CCTCATTGGAATGGAAGCAC
PanACT_Rev	qRT-PCR	TTCCAGGAAACATGGTGGAC
PanD27_For	qRT-PCR	GGATCGCCATTCAACATCTC
PanD27_Rev	qRT-PCR	GACAAGTTCCCGCTGTTTTG
PanCCD8_For	qRT-PCR	TGGCAAGATTGTGACGAGTG
PanCCD8_Rev	qRT-PCR	CCTCCCTTCTTCGTCCTTATC
PanCCD7_For	qRT-PCR	TGAAGAAGACGATGGCTACG
PanCCD7_Rev	qRT-PCR	AGTTTCGCCACAAGAGCATC
PanNSP2_Genotyping_For	PCR	ATCCTATCCCAAACCACACG
PanNSP2_Genotyping_Rev	PCR	TTGCTCCAAATCCTCCTCTG
